# Acupuncture methods for diabetic peripheral neuropathy: A protocol for a Bayesian network meta-analysis

**DOI:** 10.1097/MD.0000000000024967

**Published:** 2021-03-12

**Authors:** Hai Lun Jiang, Qiang Zhang, Yu Zheng Du, Xiang Gang Meng, Hai Peng Ban, Yang Tao Lu

**Affiliations:** a First Teaching Hospital of Tianjin University of Traditional Chinese Medicine; b Tianjin University of Traditional Chinese Medicine; c National Clinical Research Center for Chinese Medicine Acupuncture and Moxibustion, Tianjin; d BeiJing Daxing District Hospital of Integrated Chinese and Western Medicine, Beijing; e Tianjin Academy of Traditional Chinese Medicine Affiliated Hospital, Tianjin, China.

**Keywords:** acupuncture, Bayesian, diabetic peripheral neuropathy, protocol

## Abstract

**Background:**

Many clinical trials and systematic reviews have suggested that acupuncture (include moxibustion) could be effective in the treatment of diabetic peripheral neuropathy (DPN). However, clinical practices vary greatly leads to different choices which are mainly based on personal experience. The aim of this Bayesian network meta-analysis is to compare the efficacy of different acupuncture methods for DPN.

**Methods:**

Randomized controlled trials on acupuncture treatment of DPN published before January of 2021 will be searched in 9 databases including Medline, Web of Science, PubMed, Cochrane Library, Excerpta Medica Database, Sinomed, China National Knowledge Infrastructure, WanFang, and China Science and Technology Journal Database. The methodological assessment performed using the risk of bias assessment tool of Cochrane, and the level of evidence quality for the main results will be evaluated by a recommended grading, evaluation, formulation, and evaluation system approach. Bayesian network meta-analysis will be conducted using STATA V.14.0 and WinBUGS V.1.4.3.

**Results:**

The primary outcome involves: clinical efficacy. The secondary outcomes include: motor nerve conduction velocity, sensory nerve conduction velocity, Toronto clinical scoring system, Michigan neuropathy screening instrument, the modified Toronto Clinical Neuropathy Scale, the Utah early neuropathy scale, or the neuropathy disability score, and adverse reactions.

**Conclusion:**

To find the most effective acupuncture therapy for the treatment of DPN supported by evidence-based medicine.

## 1 Introduction

The 2017 International Diabetes Federation Diabetes Map estimated that by 2045, diabetes mellitus (DM) patients may reach 629 million.^[1]^ Studies have found that 10% to 20% of patients have DPN at the time when they are diagnosed with DM.^[2]^ The incidence of DPN ranges from 30% to 90% when the course of DM reaches 5 years, 10 years, or 20 years.^[3]^ The symptoms of DPN are not obvious in the early stage of onset, and it is easy to be ignored. With the development of the disease, it can cause sensory function disorders, foot ulcers and infections, and even amputation due to gangrene. Among all nontraumatic amputations, studies have found that patients with diabetic foot amputation rank first among them,^[4]^ and the mortality rate after amputation can reach up to 13%.^[5]^ DPN has not only brought great pain to the patients, but bring a huge economic burden to society. The pathogenesis of DPN is complicated and not yet clear. Modern medicine believes that DPN may be the result of a combination of various factors such as abnormal metabolism, neurotrophic factor deficiency, oxidative stress, and gene mutations caused by long-term induction of hyperglycemia. However, western medicine still lacks direct and accurate treatments for DPN, and it is necessary to further explore its standardized and effective comprehensive treatment plan.

Acupuncture is an effective treatment method in traditional Chinese medicine. The treatment of DPN by acupuncture is based on the overall concept and individualized treatment for different syndrome of traditional Chinese medicine diagnosis. It can adjust human body functions in multiple links and multiple targets, improve patients’ clinical symptoms, and it can improve related nerve conduction velocity indicators.^[6]^ Many studies has reported that acupuncture can treat DPN effectively.^[7–11]^ However, there are no Bayesian network meta-analysis has been conducted to assess which acupuncture method is the most effective for DPN patients.

## 2 Study registration

This protocol of Bayesian network meta-analysis has been drafted under the guidance of the preferred reporting items for systematic reviews and meta-analyses protocols.^[12]^

The protocol has been registered on open science framework (OSF) on December 28, 2020. (Registration number: DOI 10.17605/OSF.IO/36ZQH, https://osf.io/36zqh/)

## 3 Methods

### 3.1 Inclusion criteria

#### 3.1.1 Study type

We will select clinical randomized controlled trials published before January of 2021, and without any regional and language restrictions. Animal studies, case reports, retrospective studies, and reviews will be excluded. About duplicate articles, we will prefer to the one with more comprehensive data.

#### 3.1.2 Participants

Patients suffering from diabetic peripheral neuropathy according to the Position Statement by the American Diabetes Association^[13]^ or the Consensus on diagnosis and treatment of diabetic peripheral neuropathy^[14]^ or any other accepted diagnostic guidelines. Regardless of age, gender, type of diabetes, nationality, and disease duration.

#### 3.1.3 Interventions and comparison

The interventions for the experimental group must contains acupuncture, including one of traditional manual acupuncture, electroacupuncture, moxibustion acupuncture, fire needling, or a combination of any 2 of these acupuncture methods or combinations of any of these acupuncture methods with conventional medicinal methods. Regardless of acupoint selection or needling techniques.

The interventions for the control group is placebo, or conventional medicinal methods, or different acupunctures method from the experimental group (including combination any of these acupuncture methods with conventional medicinal methods).

### 3.2 Exclusion criteria

Trials comparing acupoint selections or acupuncture manipulations will be excluded.

### 3.3 Outcomes

#### 3.3.1 Primary outcomes: clinical efficacy

The included randomized controlled trial (RCT)s’ clinical efficacy evaluation was based on the following criteria^[15]^:

(1) Effectiveness: symptoms and/or signs of peripheral nerve dysfunction improved, and motor nerve conduction velocity or sensory nerve conduction velocity increased;
(2) Ineffectiveness: symptoms and/or signs of peripheral nerve had not improved, or motor nerve conduction velocity or sensory nerve conduction velocity did not obviously improve.

When the effect of symptoms and/or signs is inconsistent with the effect of nerve conduction velocity, the lower effective parameter is applied to show the comprehensive effect to represent the clinical efficacy.

#### 3.3.2 Secondary outcomes

Motor nerve conduction velocity, Sensory nerve conduction velocity, Toronto clinical scoring system, Michigan neuropathy screening instrument, the modified Toronto Clinical Neuropathy Scale, the Utah early neuropathy scale, or the neuropathy disability score and adverse reactions. Electrophysiological testing assisted the diagnosis of DPN. Toronto clinical scoring system, Michigan neuropathy screening instrument, modified Toronto Clinical Neuropathy Scale, Utah early neuropathy scale, neuropathy disability score are used to evaluate symptoms and/or signs. Adverse reaction involved to evaluate the safety of acupuncture therapy.^[13]^

### 3.4 Search strategy

The keywords such as “Acupuncture,” “Acupoint,” “Diabetic peripheral neuropathy,” and “Randomized Controlled Trial” were used to search in the following electronic databases: Medline, Web of Science, PubMed, Cochrane Library, Excerpta Medica Database, Sinomed, China National Knowledge Infrastructure, WanFang, and VIP databases, and there is no regional and language restrictions. We will also search for RCTs from published reviews or meta-analyses in case any literature is missed. The literature we searched will be from the beginning up to January of 2021. The search strategy for PubMed is presented in the following Table 1.

**Table 1:** OA_ Table, which illustrates search strategy of the article.

1. Acupuncture [MeSH Terms]
2. Pharmacoacupuncture [Title/Abstract]
3. Acupuncture [Title/Abstract]
4. Electroacupuncture [Title/Abstract]
5. moxibustion acupuncture [Title/Abstract]
6. warming needle moxibustion [Title/Abstract]
7. fire needling [Title/Abstract]
8. fire needle [Title/Abstract]
9. fire acupuncture [Title/Abstract]
10. OR 1–9
11. Randomized Controlled Trial [MeSH Terms]
12. Clinical Trials, Randomized [Title/Abstract])
13. Trials, Randomized Clinical [Title/Abstract]
14. Controlled Clinical, Trials, Randomized [Title/Abstract]
15. RCT [Title/Abstract]
16. Randomly [Title/Abstract]
17. placebo [Title/Abstract]
18. OR 11-16
19. Acupoint [MeSH Terms]
20. Acupuncture Point [Title/Abstract]
21. Point, Acupuncture [Title/Abstract]
22. Points, Acupuncture [Title/Abstract]
23. OR 18-21
24. Diabetic Neuropathies [MeSH Terms]
25. Neuropath^∗^, Diabetic[Title/Abstract]
26. Neuralgia^∗^, Diabetic [Title/Abstract]
27. Polyneuropath^∗^, Diabetic [Title/Abstract]
28. Mononeuropath^∗^, Diabetic [Title/Abstract]
29. Amyotroph^∗^, Diabetic [Title/Abstract]
30. OR 24-29
31. 10 AND 18 AND 23 AND 30

### 3.5 Study selection and data extraction

**Figure 1:**
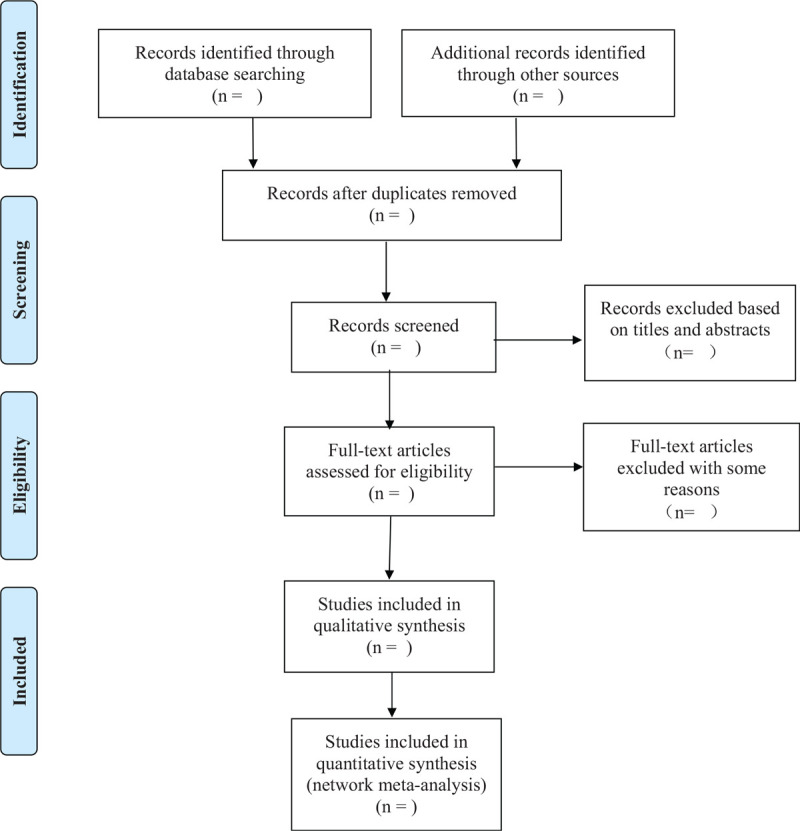
OA_Figure, which presents the flow chart of study identification and selection.

Two independent investigators will use Endnote X9 and Excel 2018 select and extract data. First, importing the retrieved literatures into EndNote X9, and then removing duplicate literatures through steps of EndNote X9. Second, we browse the titles and abstracts, and manually remove duplicates or those do not meet the inclusion criteria. Any disagreement will be resolved by discussion until consensus is reached, if opinions persisted, then the third investigator will be consulted to make the final decision. Third, 2 independent investigators will read the full-text and choose the literatures according to inclusion and exclusion criteria. If disagreements rise up and persisted after discussion, then the third investigator was consulted to make the final decision. Finally, characteristics of trials (name of author, year of publication, language, country, diagnose criteria, trial registration), characteristics of patients (age of participants, gender), details of trials (sample size, type of diabetes, duration of DPN, intervention measures, intervention time, randomization, allocation concealment method, blinding method, follow-up duration, acupoint selection, adverse events, and outcome indicators) were summarized in an Excel file. The flow chart of study identification and selection will be presented in the Figure 1.

### 3.6 Quality assessment

#### 3.6.1 Risk-of-bias assessments

Two independent investigators assessed the risk of bias, and each trial will be scored as high, low, or unclear risk based on the Cochrane Collaboration Risk of Bias Tool.^[16]^ Two independent investigators evaluate methodological quality of data covers the following 7 domains:

(1) Random sequence generation (section bias),
(2) Allocation concealment (section bias),
(3) Blinding of participants and personnel (performance bias),
(4) Blinding of outcome assessment (detection bias),
(5) Incomplete outcome data (attrition bias),
(6) Selective reporting (reporting bias),
(7) Other biases.

If a disagreement occurred regarding the risk of bias assessments of a study, the third investigator is the referee in case of disagreements.

#### 3.6.2 Quality of evidence assessments

Two independent investigators assessed the level of the quality of evidence with primary outcomes based on grading of recommendations assessment, development, and evaluation system for rating the quality of evidence and strength of recommendations.^[17]^ Grading of recommendations assessment, development, and evaluation profiler 3.6 will be used to conducted the assessment.

### 3.7 Statistical analysis

In this Bayesian network meta-analysis, STATA V.14.0 (Version 14.0; developed by the USA's StataCorp LLC) and WinBUGS V.1.4.3 (Bayesian inference Using Gibbs Sampling for Windows, Version 1.4.3; developed by UK 's Imperial College and MRC) to perform the network meta-analysis.

#### 3.7.1 Pairwise meta-analysis

STATA V.14.0 will be used to perform Pairwise meta-analysis, odds ratio were used to express dichotomous variables, whereas the standardized mean differences used to express continuous outcomes and a 95% confidence internal were used to denote both types of effect sizes. The *I*^2^ statistic will be used to assess statistical heterogeneity. When *I*^2^ < 50%, a fixed-effect model was applied. When *I*^2^ > 50%, a random effects model will be used to perform the pairwise meta-analysis, and the source of heterogeneity was analyzed. Statistical significance was set at *P* < .05.

#### 3.7.2 Network meta-analysis

WinBUGS V.1.4.3 will be used to perform Network meta-analysis, which will be conducted under a Bayesian approach using Markov Chain Monte Carlo simulation. We will use a random effects model to perform a Bayesian network meta-analysis. Iteration number will be set to 50,000, and the first 10,000 iterations for annealing will be set up to eliminate influences of the initial value.

In each closed loop, the node splitting method and the *Z*-tests will assess inconsistency of the direct and indirect evidence. For the network to be consistent, the direct and indirect effect estimates should be similar to each other.^[18]^*P* < .05 was considered evidence of network inconsistency, *P* > .05 indicate good consistency. When *P* < .05, the network inconsistencies will be reported. The summary statistical measure used was the odds ratio or standardized mean differences both with 95% credible intervals.

STATA V.14.0 will be used to plot surface under the cumulative ranking curve. The value is the percentage of the area under the curve, ranging from 0% to 100%, which will represent the ranking of therapeutic effect of acupuncture on DPN, with 100% indicating the best treatment and 0% meaning the worst.

### 3.8 Sensitivity analysis and subgroup analysis

Random effects network meta-regression model will be used to perform a network meta-regression in order to explore the source of heterogeneity.

In order to obtain a stable conclusion of network meta-analysis, we will conduct a sensitivity analysis, to check whether there are abnormal data caused the bias. If there are such data, grouping them and then analyzing or discussing them will be considered.

If the necessary data are available, subgroup analysis will be carried out according to different factors as follows: interventions (duration of treatment, period of treatment), participant characteristics (geographical region, race, nationality, age, gender, type of diabetes, duration of disease) and the type of intervention in the control group.

### 3.9 Publication bias assessment

Egger test was performed to evaluate the publication bias of the primary outcome. When *P > *.05, the result of Egger test revealed no publication bias, conversely; when *P* < .05, indicates that it may exist bias.

### 3.10 Additional analyses

We try to minimize clinical heterogeneity as possible, however, the clinical heterogeneity caused by different acupuncturists is unavoidable, especially in acupoint selection and the amount of stimulation. If quantitative synthesis is not appropriate, we will conduct a descriptive analysis of the study which may generate heterogeneity in the treatment outcomes.

## 4 Discussion

Acupuncture therapies to treat DPN are easily accepted by the majority of patients due to avoiding the serious side effects of chemical drugs.

Multiple RCTs have demonstrated the efficacy of acupuncture in the treatment of DPN, however, the procedure of acupuncture treatment on DPN has not yet been standardized and the quality of the clinical trials were uneven, as a result, clinicians tend to choose acupuncture therapies based on their own clinical experience rather than the most effective acupuncture treatment which is supported by evidence-based medicine. Network meta-analysis can evaluate and sequence a variety of different acupuncture therapies directly or indirectly.^[19]^ To the best of our knowledge, this study will be the first network meta-analysis of acupuncture methods for the treatment of DPN. The results of this study may be useful to clinicians, practice guide developers, researchers, decision makers, and so on. Therefore, we hope that our results will provide credible evidence to support the clinical selection of acupuncture and encourage wider acceptance of acupuncture as complementary and alternative medicine for DPN.

## 5 Conclusion

Based on current RCTs, our study will compare the efficacy of different acupuncture methods for DPN and develop a proposal for acupuncture-based treatment for DPN.

## Author contributions

**Data curation:** Xianggang Meng, Yang Tao Lu.

**Project administration:** Yuzheng Du.

**Software:** Haipeng Ban.

**Writing – original draft:** Hailun Jiang, Qiang Zhang.

**Writing – review & editing:** Hailun Jiang.
